# Insights into the bacterial symbiont diversity in spiders

**DOI:** 10.1002/ece3.4051

**Published:** 2018-04-19

**Authors:** Lihua Zhang, Yueli Yun, Guowen Hu, Yu Peng

**Affiliations:** ^1^ Centre for Behavioral Ecology and Evolution College of Life Sciences Hubei University Wuhan China; ^2^ Hubei Collaborative Innovation Center for Green Transformation of Bio‐Resources College of Life Sciences Hubei University Wuhan China

**Keywords:** Araneae order, bacterial community, co‐infection, endosymbionts, high‐throughput sequencing

## Abstract

Most spiders are natural enemies of pests, and it is beneficial for the biological control of pests to learn the relationships between symbionts and their spider hosts. Research on the bacterial communities of insects has been conducted recently, but only a few studies have addressed the bacterial communities of spiders. To obtain a complete overview of the microbial communities of spiders, we examined eight species of spider (*Pirata subpiraticus*,* Agelena difficilis*,* Artema atlanta*,* Nurscia albofasciata*,* Agelena labyrinthica*,* Ummeliata insecticeps*,* Dictis striatipes,* and *Hylyphantes graminicola*) with high‐throughput sequencing based on the V3 and V4 regions of the 16S rRNA gene. The bacterial communities of the spider samples were dominated by five types of endosymbionts, *Wolbachia*,* Cardinium*,* Rickettsia*,* Spiroplasma,* and *Rickettsiella*. The dominant OTUs (operational taxonomic units) from each of the five endosymbionts were analyzed, and the results showed that different spider species were usually dominated by special OTUs. In addition to endosymbionts, *Pseudomonas*,* Sphingomonas*,* Acinetobacter*,* Novosphingobium*,* Aquabacterium*,* Methylobacterium*,* Brevundimonas*,* Rhizobium*,* Bradyrhizobium*,* Citrobacter*,* Arthrobacter*,* Pseudonocardia*,* Microbacterium*,* Lactobacillus,* and *Lactococcus* were detected in spider samples in our study. Moreover, the abundance of *Sphingomonas*,* Methylobacterium*,* Brevundimonas,* and *Rhizobium* in the spider *D. striatipes* was significantly higher (*p *<* *.05) than the bacterial abundance of these species in seven other spider species. These findings suggest that same as in insects, co‐infection of multiple types of endosymbionts is common in the hosts of the Araneae order, and other bacterial taxa also exist in spiders besides the endosymbionts.

## 1 INTRODUCTION

Most arthropods are infected with a variety of symbiotic bacteria, which generally affect their hosts in a number of ways, such as impacting on development, reproduction, and speciation (Brucker & Bordenstein, 2012; Duron et al., 2008; Goodacre, Martin, Thomas, & Hewitt, 2006; McFall‐Ngai, 2002), providing protection against natural enemies and pathogens (Oliver, Russell, Moran, & Hunter, 2003; Scarborough, Ferrari, & Godfray, 2005), supplying key nutrients (Brownlie et al., 2009; Douglas, 1998) and improving heat tolerance (Montllor, Maxmen, & Purcell, 2002). To provide insights into bacterial communities and the relationships between symbiotic bacteria and their hosts, research regarding bacterial diversity and bacterial communities within insects has recently increased (Bili et al., 2016; Monteiro et al., 2016; Muturi, Ramirez, Rooney, & Dunlap, 2016; Zhao et al., 2016). Spiders are perceived as important natural enemies for pests (Marc, Canard, & Ysnel, 1999; Nyffeler & Sunderland, 2003), and many researchers have focused their attention on the endosymbionts infection of spiders (Duron et al., 2008; Goodacre et al., 2006; Rowley, Raven, & McGraw, 2004) and the relationships between the endosymbionts (such as *Wolbachia*,* Cardinium*,* Rickettsia* and *Spiroplasma*) and their spider hosts (Curry, 2013; Gunnarsson, Goodacre, & Hewitt, 2009; Martin & Goodacre, 2009). In regard to the bacterial community of spiders, only a few studies have been reported (Vanthournout & Hendrickx, 2015; Zhang, Zhang, Yun, & Peng, 2017). Vanthournout & Hendrickx detected the bacterial community of a dwarf spider, *Oedothorax gibbosus*, and the study suggested that endosymbionts (such as *Rhabdochlamydia*,* Cardinium*,* Wolbachia,* and *Rickettsia*) dominated the bacterial communities in this spider. Zhang et al. tested the microbial community of spider *Marpiss magister*, and both endosymbionts and other bacteria (suspected as gut bacteria or environmental bacteria) were detected.

Comparing the prevailing research on the bacterial community of insects, the bacterial communities of only single spider species have been examined. To provide insights into the bacterial diversity of multiple spiders (especially the bacteria not belonging to endosymbionts), in this study, we detected the bacterial diversity of eight spider species using a high‐throughput sequencing technique, and through the distribution and relative abundance of different bacteria in different spider species, we analyzed the difference in bacterial communities among all eight spider species. By revealing the other bacteria (besides endosymbionts) in spiders, this research on the symbionts of spiders will add to the understanding of all bacteria (such as gut bacteria or environmental bacteria) besides common endosymbionts.

## 2 MATERIALS AND METHODS

### 2.1 Sample collection

In the summer of 2016, three species of spiders (*Pirata subpiraticus*,* Agelena difficilis,* and *Artema Atlanta*) were collected near Shahu, Wuhan (China), and three species of spiders (*Nurscia albofasciata*,* Agelena labyrinthica*,* and Ummeliata insecticeps*) were collected near Shizishan, Wuhan (China). *Dictis striatipes* was collected in Guangpo, Lingshui (China), and *Hylyphantes graminicola* was collected in Longmen, Luoyang (China; See Table 1). Ten individuals were collected for each spider species, and all spiders collected in this study were ecologically important species (Zhang & Wang, 2017). The species were identified based on the morphological features of the specimens. Living samples were transported to the laboratory and starved for 2 weeks. Then, samples were fixed in 100% ethanol and stored at −20°C. All eight spider species used in this study were identified as a nonendangered and nonprotected species.

**Table 1:** Spider samples used in this study

Species	Genera	Family	Location	Group of DNA pools (anumbers)
*P. subpiraticus*	*Pirata*	Lycosidae	Shahu, Wuhan	D (5)
*N. albofasciata*	*Nurscia*	Titanoecidae	Shizishan, Wuhan	K (5)
*D. striatipes*	*Dictis*	Scytodidae	Guangpo, Lingshui	M (4)
*A. labyrinthica*	*Agelena*	Agelenidae	Shizishan, Wuhan	O (5)
*A. difficilis*	*Agelena*	Agelenidae	Shahu, Wuhan	P (4)
*A. atlanta*	*Artema*	Pholcidae	Shahu, Wuhan	R (5)
*U. insecticeps*	*Ummeliata*	Linyphiidae	Shizishan, Wuhan	S (4)
*H. graminicola*	*Hylyphantes*	Linyphiidae	Longmen, Luoyang	T (5)

a The number of DNA pools in each spider species.

### 2.2 DNA extraction

Each sample was cleaned using an ultrasonic cleaner (FRQ‐1004T) filled with a 75% alcoholic solution for 1–2 min to remove surface bacteria and pollutants, followed by three washes with sterile ultrapure water. The DNA was extracted from each individual (whole body) using the QIAGEN DNeasy Kit (Germany) following the manufacturer's recommended protocol. DNA was then quantified using a nanophotometer (NanoPhotometer NP80 Touch, Implen GmbH). An equimolar amount of DNA from each of the two individuals of the same species was mixed into one of the DNA pools. The name of the DNA groups and the numbers of DNA pools in each spider species are shown in Table 1.

### 2.3 Amplification and sequencing

Each pooled DNA sample was amplified for the presence of bacteria using universal 16S rRNA gene primers (27F 5′‐AGAGTTTGATCATGGCTCAG‐3′ and 1487R 5′‐TACCTTGTTACGACTTCACC‐3′; Heddi, Grenier, Khatchadourian, Charles, & Nardon, 1999). The PCR amplification was conducted in a volume of 30 μl containing 1 μl of each primer, 0.5 μl of Taq DNA polymerase, 1 μl of dNTPs, 3 μl of 10× buffer, 0.5 μl of template DNA, and 23 μl of sterile distilled water. The following condition was used for the PCR reactions: denaturation for 5 min at 94°C, 35 cycles of denaturation for 30 s at 94°C, annealing for 45 s at 53°C, and elongation at 72°C for 45 s. For the last cycle, the elongation time was extended to 7 min at 72°C. PCR products were run on 2% agarose gels, and the samples producing visualized amplicons were utilized for high‐throughput sequencing of microbial diversity. The variable region V3–V4 of the 16S rDNA was used to assess bacterial diversity (Caporaso et al., 2012). The sequencing was conducted on an Illumina HiSeq platform at BioMarKer Technologies Co. Ltd. (Beijing, China).

### 2.4 Bioinformatic analyses

Paired‐end reads were merged into single, longer sequences using FLASH version 1.2.7 (Magoč & Salzberg, 2011). Quality filtering on the raw tags was performed under specific filtering conditions (The Sliding Window uses 50 bp. This works by scanning from 5′ end of the read and removes the 3′ end of the read when average quality of a group of bases drops below 20 bp to obtain high‐quality clean tags by Trimmomatic version 0.33 (Bolger, Lohse, & Usadel, 2014). UCHIME version 4.2 (default setting: 80% similarity) was used to identify and eliminate chimeric sequences (Edgar, Haas, Clemente, Quince, & Knight, 2011). The remaining sequences were assigned into operational taxonomic units (OTUs) at 97% similarity using UCLUST version 1.2.22 (Edgar, 2010). The taxonomic identification of each OTU was conducted by comparing the representative sequences (the sequences which has the most highest relative abundance) of each cluster against SILVA by a BLASTn search (Quast et al., 2013), and the taxonomic classification of each OTU was performed using Ribosomal Database Project (RDP) Classifier version 2.2 with the classification threshold set at 0.8 (Cole et al., 2009). The raw reads have been submitted to the NCBI Sequence Read Archive (SRA) database (Accession number: SRP132570).

Beta diversity was used to test the difference in bacterial communities between the different host species. The principal coordinate analysis (PCoA) with the Bray‐Curtis distance algorithm was performed using QIIME.

### 2.5 Statistical analyses

Differences in the relative abundance of certain bacterial types among different groups were analyzed with the Mann–Whitney *U* test.

## 3 RESULTS

### 3.1 Bacterial diversity across hosts

A total of 3,366,927 raw reads were yielded. After quality filtering and the removal of chimeric sequences, 3,137,625 sequences were retained, with a mean of 84,800 reads per sample. In total, all of the sequences were classified into 513 operational taxonomic units (OTUs) at 97% sequence identity, which belonged to 139 families and 18 phyla.

**Figure 1:**
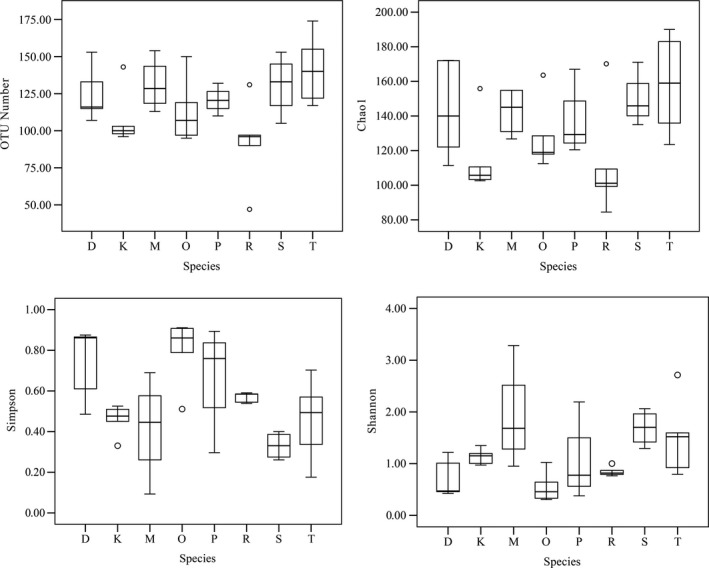
Diversity measurements of the bacterial communities of eight spider species. D, K, M, O, P, R, S, and T indicate spider species *P. subpiraticus*,* N. albofasciata*,* D. striatipes*,* A. labyrinthica*,* A. difficilis*,* A. atlanta*,* U. insecticeps,* and *H. graminicola*, respectively

**Figure 2:**
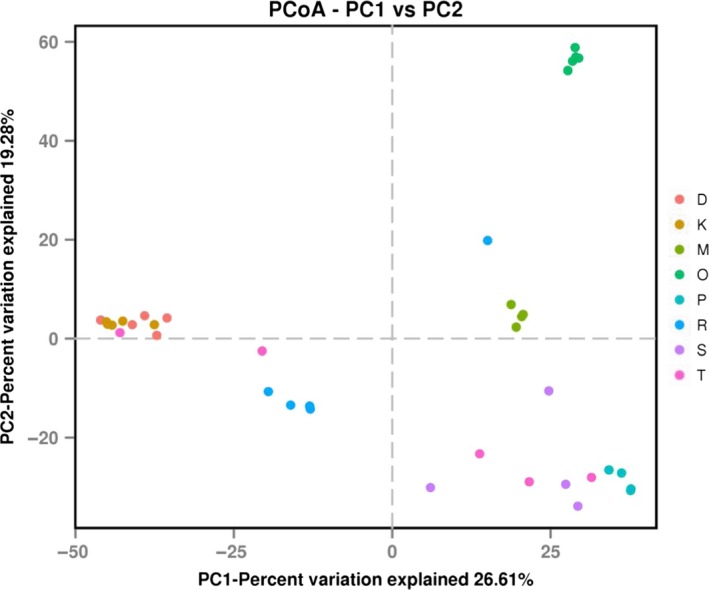
Comparison of the bacterial community structures in different species. Principal coordinate analysis was generated using the Bray‐Curtis distance between the bacterial communities for each analyzed sampled. Different colors represent different species. D, K, M, O, P, R, S, and T indicate spider species *P. subpiraticus*,* N. albofasciata*,* D. striatipes*,* A. labyrinthica*,* A. difficilis*,* A. atlanta*,* U. insecticeps,* and *H. graminicola*, respectively

The present spider species had a high number of OTUs ranging between 92.20 ± 13.40 and 141.60 ± 10.54. *H. graminicola* had the highest number of identified OTUs compared to that of other spider species. The predicted number of OTUs (Chao 1) exceeds those observed in the present spider species. The Chao 1 index value for *N. albofasciata* was significantly lower than that for *H. graminicola* (*p *<* *.05). The Simpson index value for *A. labyrinthica* was significantly higher than that for *U. insecticeps*,* N. albofasciata*,* D. striatipes* and *A. labyrinthica* (*p *<* *.05). The Shannon index value for *A. labyrinthica* was significantly lower than that for *U. insecticeps*,* N. albofasciata*,* D. striatipes,* and *A. labyrinthica* (*p *<* *.05). All of the data showed that the microbiota of the present spider species had a high diversity (Figure 1). The dissimilarity between the bacterial communities of samples was quantified by the Bray‐Curtis distance. Principal coordinate analysis (PCoA) showed that the bacterial communities were much more similar within species than between species (Figure 2).

### 3.2 The endosymbiont composition of bacterial communities in the spider hosts

The bacterial diversity of spiders in this study reached 270 genera; however, bacterial communities in the samples were mostly dominated by endosymbionts such as *Wolbachia*,* Cardinium*,* Rickettsia*,* Spiroplasma,* and *Rickettsiella*. *Wolbachia* was the most dominant symbiotic bacteria in *P. subpiraticus* (83.92 ± 6.25%), *N. albofasciata* (60.56 ± 4.22%), and *H. graminicola* (51.43 ± 20.08%). *Wolbachia* was also detected in *A. atlanta and U. insecticeps*. *Rickettsia* was detected in *A. atlanta* (58.90 ± 12.36%) and *U. insecticeps* (25.29 ± 14.67%) with high relative abundances. *Rickettsiella* was the most abundant group in *A. labyrinthica* (87.58 ± 5.58%). It was also composed of a large proportion in *N. albofasciata* and *A. atlanta* and accounted for 25.29 ± 2.33% and 17.60 ± 17.59% of the microbe. The most dominant bacterial symbiont in *D. striatipes* was *Spiroplasma*, which composed 58.29 ± 13.47% of the total microbe. *Cardinium* dominated the bacterial communities in *H. graminicola*,* U. insecticeps,* and *A. difficilis* and accounted for 36.98 ± 6.62% ~ 79.96 ± 9.70% (see Table 2).

**Table 2:** Composition and abundance of the top 20 symbiotic bacterial types in the bacterial community of eight spider species

Taxon	D	K	M	O	P	R	S	T	*p*‐Value
**Proteobacteria**	91.62 ± 6.31	99.21 ± 0.10	34.64 ± 11.99	98.86 ± 0.21	13.86 ± 5.68	99.35 ± 0.23	43.73 ± 9.32	67.98 ± 14.46	.000
*Wolbachia*	83.92 ± 6.25	60.56 ± 4.22	0.01 ± 0.00	0.01 ± 0.00	0.02 ± 0.01	19.42 ± 4.36	6.55 ± 6.51	51.43 ± 20.08	.001
*Rickettsiella*	0.01 ± 0.00	25.29 ± 2.33	0.00 ± 0.00	87.58 ± 5.58	0.01 ± 0.00	17.60 ± 17.59	0.01 ± 0.00	0.00 ± 0.00	.001
*Rickettsia*	0.00 ± 0.00	6.16 ± 6.15	0.00 ± 0.00	0.01 ± 0.01	0.00 ± 0.00	58.90 ± 12.36	25.29 ± 14.67	5.62 ± 5.55	.003
*Pseudomonas*	0.44 ± 0.27	0.17 ± 0.01	2.07 ± 1.37	0.19 ± 0.03	0.79 ± 0.59	0.17 ± 0.08	0.80 ± 0.47	0.77 ± 0.30	.069
*Sphingomonas*	0.35 ± 0.09	0.28 ± 0.01	4.67 ± 1.07	0.31 ± 0.06	0.59 ± 0.20	0.22 ± 0.08	0.44 ± 0.18	0.70 ± 0.39	.063
*Acinetobacter*	0.32 ± 0.15	0.10 ± 0.04	0.98 ± 0.81	0.11 ± 0.05	0.23 ± 0.10	0.07 ± 0.04	1.15 ± 0.38	0.37 ± 0.29	.039
*Novosphingobium*	0.40 ± 0.18	0.19 ± 0.03	0.79 ± 0.20	0.14 ± 0.02	0.61 ± 0.26	0.09 ± 0.03	0.30 ± 0.13	0.32 ± 0.17	.006
*Aquabacterium*	0.22 ± 0.08	0.14 ± 0.03	0.69 ± 0.15	0.16 ± 0.04	0.45 ± 0.22	0.10 ± 0.03	0.33 ± 0.11	0.29 ± 0.13	.021
*Methylobacterium*	0.10 ± 0.05	0.05 ± 0.02	1.24 ± 0.21	0.11 ± 0.06	0.15 ± 0.08	0.03 ± 0.0.01	0.07 ± 0.06	0.11 ± 0.07	.031
*Brevundimonas*	0.05 ± 0.04	0.04 ± 0.01	2.42 ± 1.61	0.04 ± 0.01	0.10 ± 0.06	0.02 ± 0.01	0.13 ± 0.08	0.12 ± 0.07	.018
*Rhizobium*	0.02 ± 0.02	0.02 ± 0.01	2.00 ± 0.84	0.01 ± 0.00	0.02 ± 0.01	0.01 ± 0.01	0.03 ± 0.01	0.03 ± 0.01	.032
*Citrobacter*	0.01 ± 0.00	0.00 ± 0.00	1.77 ± 1.75	0.00 ± 0.00	0.01 ± 0.01	0	0.02 ± 0.01	0.04 ± 0.03	.265
*Bradyrhizobium*	0.05 ± 0.01	0.10 ± 0.02	0.43 ± 0.11	0.06 ± 0.01	0.12 ± 0.04	0.06 ± 0.02	0.09 ± 0.02	0.10 ± 0.06	.069
**Bacteroidetes**	0.29 ± 0.04	0.10 ± 0.02	1.66 ± 0.73	0.11 ± 0.05	80.27 ± 9.58	0.05 ± 0.03	37.41 ± 6.94	29.53 ± 13.82	.000
*Cardinium*	0.00 ± 0.00	0.00 ± 0.00	0.00 ± 0.00	0.01 ± 0.01	79.96 ± 9.70	0.00 ± 0.00	36.98 ± 6.62	29.29 ± 13.81	.000
**Tenericutes**	6.38 ± 6.38	0.00 ± 0.00	58.29 ± 13.47	0.00 ± 0.00	0.00 ± 0.00	0.01 ± 0.00	14.21 ± 14.20	0.00 ± 0.00	.045
*Spiroplasma*	6.38 ± 6.38	0.00 ± 0.00	58.29 ± 13.47	0.00 ± 0.00	0.00 ± 0.00	0.01 ± 0.00	14.21 ± 14.20	0.00 ± 0.00	.045
**Actinobacteria**	1.22 ± 0.28	0.48 ± 0.09	4.26 ± 0.46	0.70 ± 0.16	3.98 ± 2.62	0.33 ± 0.09	0.87 ± 0.33	1.42 ± 0.80	.006
*Arthrobacter*	0.40 ± 0.12	0.15 ± 0.02	1.01 ± 0.27	0.23 ± 0.05	0.87 ± 0.60	0.17 ± 0.05	0.28 ± 0.07	0.44 ± 0.20	.045
*Pseudonocardia*	0.01 ± 0.01	0.10 ± 0.09	1.24 ± 0.35	0.00 ± 0.00	1.03 ± 0.72	0.00 ± 0.00	0.07 ± 0.07	0.07 ± 0.05	.007
*Microbacterium*	0.10 ± 0.02	0.03 ± 0.01	0.25 ± 0.12	0.07 ± 0.06	0.28 ± 0.23	0.01 ± 0.00	0.12 ± 0.09	0.13 ± 0.08	.009
**Firmicutes**	0.28 ± 0.23	0.05 ± 0.02	0.24 ± 0.07	0.19 ± 0.09	1.30 ± 1.16	0.14 ± 0.10	3.54 ± 3.44	0.62 ± 0.38	.467
*Lactobacillus*	0.09 ± 0.08	0.01 ± 0.00	0.06 ± 0.01	0.05 ± 0.04	0.45 ± 0.45	0.04 ± 0.03	1.86 ± 1.86	0.01 ± 0.01	.273
*Lactococcus*	0.12 ± 0.12	0.00 ± 0.00	0	0.00 ± 0.00	0.48 ± 0.48	0.03 ± 0.03	1.51 ± 1.51	0	.838

Data were shown as the mean ± *SE*. The data were compared using a nonparametric Kruskal–Wallis test, which tests for differences between different groups. The relative abundance of each bacterial taxa was tested using the significant difference between groups when *p* < .05. D, K, M, O, P, R, S, and T indicate spider species *P. subpiraticus*,* N. albofasciata*,* D. striatipes*,* A. labyrinthica*,* A. difficilis*,* A. atlanta*,* U. insecticeps,* and *H. graminicola*, respectively.

The shaded and bold values indicate that the relative abundance of *Sphingomonas*,* Methylobacterium*,* Brevundimonas*, and *Rhizobium* in spider *D. striatipes* was significantly higher (*p* < .05) than the bacterial abundance in seven other kinds of spiders

### 3.3 The dominant endosymbiont OTUs in spiders

**Figure 3:**
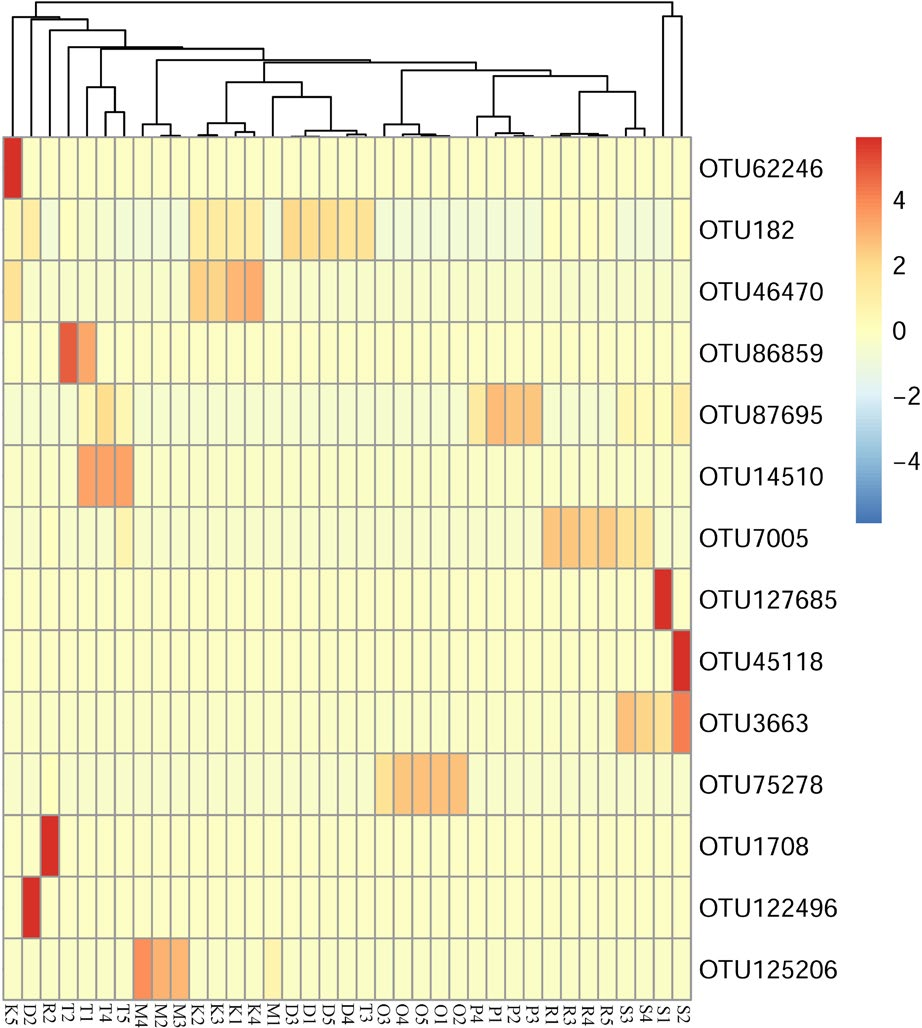
Heat map for operational taxonomic unit types in different samples

Different OTU types of endosymbionts prevailed in different spider hosts (See Figure 3, Table S1). OTU182 of *Wolbachia* was the dominant OTU type within *P. subpiraticus* (relative abundance: 61.89%~93.00%), *N. albofasciata* (relative abundance: 44.79%~68.49%), and *A. atlanta* (relative abundance: 2.24%~26.17%). OTU46470 and OTU75278 of *Rickettsiella* were the most popular microbes within *N. albofasciata* (relative abundance: 18.62%~32.04%) and *A. labyrinthica* (relative abundance: 65.81%~95.43%), respectively. OTU125206 of *Spiroplasma* prevailed in *D. striatipes* (relative abundance: 19.85%~82.82%). OTU87695 of *Cardinium* was the most dominant strain in *A. labyrinthica* (relative abundance: 51.38%~94.49%). OTU7005 of *Rickettsia* was dominant in *A. atlanta* (relative abundance: 9.55%~73.52%). For *U. insecticeps* and *H. graminicola*, there were at least two dominant OTU types of *Wolbachia* (OTU182 and OTU86859) and *Cardinium* (OTU3663 and OTU87695).

### 3.4 The other bacterial taxa of bacterial communities in spider hosts

In addition to endosymbionts, there were other bacteria in the bacterial communities of spiders. The bacteria of the phylum Proteobacteria, such as *Pseudomonas*,* Sphingomonas*,* Acinetobacter*,* Novosphingobium*,* Aquabacterium*,* Methylobacterium*,* Brevundimonas*,* Rhizobium,* and *Bradyrhizobium,* were detected in all eight spider species, and *Citrobacter* existed in seven spider species except *A. atlanta*. Bacteria from the phyla Actinobacteria, such as *Arthrobacter*,* Pseudonocardia,* and *Microbacterium*, were found in all of the samples in our study. *Lactobacillus* and *Lactococcus*, which belong to the phylum Firmicutes, were detected in spiders except *D. striatipes* and *H. graminicola* (Table 2). Moreover, the abundance of *Sphingomonas*,* Methylobacterium*,* Brevundimonas,* and *Rhizobium* in spider *D. striatipes* was significantly higher (*p *<* *.05) than the bacterial abundance in seven other kinds of spiders, and no differences were obtained for these four bacteria between the other seven spider species (Table S2).

## 4 DISCUSSION

The bacterial community of a single kind of spider has been previously conducted (Vanthournout & Hendrickx, 2015; Zhang et al., 2017), but there have been no reports regarding the research of bacterial communities in multiple spider species until now. This study tested the bacterial communities of eight spider species. Both the endosymbionts and other bacteria were detected inside the body of the spiders. Moreover, this study analyzed the distribution and relative abundance of the endosymbionts and other bacteria in different spider hosts, and the results suggest that the distribution of symbionts in different hosts displayed diversity; the endosymbiont types of co‐infection in different spiders were different; and certain other bacteria, such as *Sphingomonas*,* Methylobacterium*,* Brevundimonas,* and *Rhizobium*, in spider *D. striatipes* were significantly higher (*p *<* *.05) than these bacterial abundances in seven other kinds of spiders. This is the first report examining the bacterial communities in multiple spider species.

Co‐infection of multiple endosymbionts in the arthropods host was common (Duron et al., 2008; Engelstädter & Hurst, 2009; Goodacre et al., 2006), and relatively few studies have explored the phenotypic effect of multiple endosymbionts on their hosts (Curry, Paliulis, Welch, Harwood, & White, 2015; White, Kelly, Cockburn, Perlman, & Hunter, 2011). Little information has been obtained in relation to the emulative distribution of multiple endosymbionts in their hosts. In this study, *Wolbachia*,* Cardinium*,* Spiroplasma*,* Rickettsia,* and *Rickettsiella* were the dominant endosymbionts in spiders, but the relative abundance of each endosymbiont in different spider hosts was different (See Table 2), and the co‐infection of endosymbionts varied in different spider hosts. From our results (Table 2), *Spiroplasma* was the most dominant endosymbiont in the spider *D. striatipes*, and besides *Spiroplasma*, the relative abundance of other endosymbionts (*Wolbachia*,* Cardinium*,* Rickettsia,* and *Rickettsiella*) present in this spider was much lower (0.00 ± 0.00% ~ 0.01 ± 0.00%) than that of the dominant symbiont. A similar phenomenon of one endosymbiont dominating the host was found both in *A. labyrinthica* and *A. difficilis*. One kind of *Rickettsiella* dominated the bacterial community of *A. labyrinthica*, and the relative abundance of other endosymbionts (0.00 ± 0.00% ~ 0.01 ± 0.01%) was much less than that of *Rickettsiella*. Moreover, one kind of *Cardinium* dominated the bacterial community of *A. difficilis*, but the distribution of other endosymbionts in this spider was scarce (0.00 ± 0.00% ~ 0.02 ± 0.01%). From Figure 3, we can see that OTU125206 was the predominant OTU type in *D. striatipes*, OTU75278 was the dominant OTU type in *A. labyrinthica*, and OTU87695 was dominant in *A. difficilis*. According to our results, we supposed that a special OTU type or strain of one endosymbiont would affect the distribution of other endosymbionts in the same spider host. In contrast, there were OTU types or strains of endosymbionts that would not affect the existence of other endosymbionts in the same host.

In regard to the bacterial community of spiders, Vanthournout and Hendrickx (2015) tested the bacterial community of a dwarf spider, *Oedothorax gibbosus*, and they found that endosymbionts were the dominant bacteria in the communities, and no other bacteria were reported in their research. Zhang et al. (2017) detected the bacterial community of one kind of spider, *Marpiss magister*, and they found that other bacteria existed inside the body of the spider besides endosymbionts. In our study, not only many other bacteria (such as *Pseudomonas*,* Sphingomonas*,* Acinetobacter*,* Novosphingobium*,* Aquabacterium*,* Methylobacterium*,* Brevundimonas*,* Rhizobium*,* Bradyrhizobium*,* Arthrobacter*,* Pseudonocardia*,* Microbacterium*,* Citrobacter*,* Lactobacillus,* and *Lactococcus*) were detected in the communities of spider samples besides endosymbionts, but also the differences of bacterial abundance for these bacteria in the different spider species were analyzed. Our results showed that the abundance of endosymbionts and other bacteria inside the bodies of different spider species was different. It is well‐known that research on gut bacteria of invertebrate and vertebrate animals is increasing because of their potential function on their hosts (Berasategui et al., 2017; Sanchez‐Alcoholado et al., 2017; Smith, Srygley, Healy, Swaminath, & Mueller, 2017). Spiders have a special feeding mode. They usually bite part of the prey and then quickly inject venom into the body of prey and sucked the prey (Foelix, 2011). We hypothesize that the gut bacteria of spiders may be distinct from insects or other species in Arachnoidea (such as mites and scorpions). However, there have been no reports regarding the gut bacteria communities of spiders until now. Almost all of the nonendosymbionts present (except *Pseudonocardia*) in our study were detected in the gut of some insects (Anjum et al., 2017; Gupta et al., 2014; Snyman, Gupta, Bezuidenhout, Claassens, & van den Berg, 2016; Wang, Gilbreath, Kukutla, Yan, & Xu, 2011). Moreover, the bacteria from genus *Pseudomonas*,* Citrobacter* and *Lactococcus* were also found in the gut of a predatory beetle *Poecilus chalcites* (Lehman, Lundgren, & Petzke, 2009) Also as a kind of predator, the gut bacteria of spiders may be similar with the gut bacterial structure of predatory insects. From our results, we suppose that the gut bacteria of spiders may be composed by indigenous bacteria and environmental bacteria, and the relative abundance of bacteria within their hosts is related to the hosts’ species and the environment of the hosts.

## CONFLICT OF INTEREST

All of the authors declare that they have no conflict of interest in the publication.

## AUTHOR CONTRIBUTIONS

YUELI YUN and YU PENG designed the experiments. LIHUA ZHANG and GUOWEN HU conducted the experiments and data analysis. YUELI YUN and LIHUA ZHANG wrote the manuscript.

## DATA ACCESSIBILITY

The original data of the symbionts relative abundance in spiders are available from the Dryad Digital Repository: https://doi.org/10.5061/dryad.7k702.
