# Supplementary material for: Insights into the bacterial symbiont diversity in spiders
Source: Ecol Evol. 2018 Apr 19;8(10):4899–906. doi: 10.1002/ece3.4051 (PMC5980269; doi:10.1002/ece3.4051)
Supplement: Supplementary file 1 [file ECE3-8-4899-s001.docx]

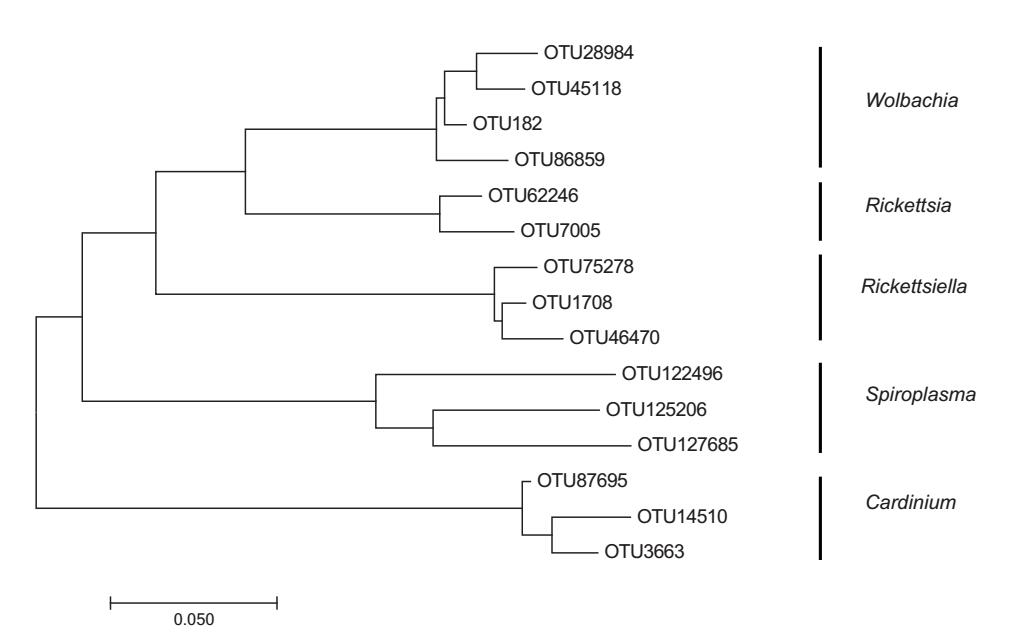


Fig. S1 the phylogenetic tree of the dominated OTUs

| S* | *Wolbachia*** | | *Rickettsiella*** | | *Cardinium*** | | *Rickettsia*** | | *Spiroplasma*** | |
| --- | --- | --- | --- | --- | --- | --- | --- | --- | --- | --- |
|  | N | Types of OTUs | N | Types of OTUs | N | Types of OTUs | N | Types of OTUs | N | Types of OTUs |
| D1 | 3 | OTU182(93.00%) |  |  | 1 |  | 1 |  |  |  |
| D2 | 4 | OTU182(61.89%) | 1 |  |  |  | 1 |  | 1 | OTU122496(31.9%) |
| D3 | 3 | OTU182(93.50%) | 1 |  | 1 |  |  |  |  |  |
| D4 | 4 | OTU182(77.60%) | 2 |  |  |  | 1 |  | 1 |  |
| D5 | 3 | OTU182(92.75%) | 1 |  |  |  |  |  | 1 |  |
| K1 | 3 | OTU182(65.28%) | 2 | OTU46470(28.79%) |  |  | 1 |  | 1 |  |
| K2 | 3 | OTU182(64.72%) | 3 | OTU46470(23.57%) | 1 |  | 2 |  | 1 |  |
| K3 | 4 | OTU182(68.49%) | 2 | OTU46470(23.42%) |  |  | 2 |  | 1 |  |
| K4 | 4 | OTU182(58.86%) | 1 | OTU46470(32.04%) | 1 |  | 1 |  | 1 |  |
| K5 | 2 | OTU182(44.79%) | 2 | OTU46470(18.62%) |  |  | 2 | OTU62246(30.74%) |  |  |
| M1 | 1 |  | 1 |  | 1 |  | 1 |  | 1 | OTU125206(19.85%) |
| M2 | 2 |  | 1 |  |  |  |  |  | 2 | OTU125206(66.45%) |
| M3 | 1 |  | 2 |  |  |  | 1 |  | 1 | OTU125206(64.02%) |
| M4 | 1 |  | 3 |  | 1 |  | 2 |  | 2 | OTU125206(82.82%) |
| O1 | 1 |  | 2 | OTU75278(95.43%) |  |  |  |  | 2 |  |
| O2 | 2 |  | 3 | OTU75278(95.25%) | 1 |  |  |  |  |  |
| O3 | 1 |  | 3 | OTU75278(65.81%) | 2 |  | 1 |  | 1 |  |
| O4 | 1 |  | 2 | OTU75278(88.68%) | 2 |  | 1 |  | 1 |  |
| O5 | 2 |  | 1 | OTU75278(92.71%) | 1 |  |  |  |  |  |
| P1 | 2 |  | 2 |  | 1 | OTU87695(94.49%) |  |  | 1 |  |
| P2 | 4 |  | 2 |  | 2 | OTU87695(88.24%) | 1 |  |  |  |
| P3 | 2 |  | 1 |  | 4 | OTU87695(85.73%) | 1 |  | 1 |  |
| P4 | 1 |  | 1 |  | 1 | OTU87695(51.38%) |  |  |  |  |
| R1 | 3 | OTU182(24.34%) | 1 |  | 1 |  | 1 | OTU7005(72.5%) | 1 |  |
| R2 | 1 | OTU182(2.24%) | 3 | OTU1708(74.65%) | 1 |  | 1 | OTU7005(9.55%) | 1 |  |
|  |  |  |  | OTU75278(13.31%) |  |  |  |  |  |  |
| R3 | 4 | OTU182(22.3%) | 2 |  | 1 |  | 1 | OTU7005(73.52%) |  |  |
| R4 | 4 | OTU182(26.17%) | 2 |  |  |  | 1 | OTU7005(68.95%) | 2 |  |
| R5 | 3 | OTU182(21.89%) | 3 |  | 1 |  | 2 | OTU7005(69.95%) | 1 |  |
| S1 | 2 |  | 2 |  | 8 | OTU3663(3.77%) | 1 |  | 3 | OTU127685(56.80%) |
|  |  |  |  |  |  | OTU87695(19.13%) |  |  |  |  |
| S2 | 10 | OTU182(23.66%) | 2 |  | 8 | OTU3663(8.68%) |  |  |  |  |
|  |  | OTU45118(1.18%) |  |  |  | OTU87695(43.76%) |  |  |  |  |
| S3 | 2 |  | 2 |  | 12 | OTU3663(6.27%) | 1 | OTU7005(54.09%) | 1 |  |
|  |  |  |  |  |  | OTU87695(32.08%) |  |  |  |  |
| S4 | 4 |  | 3 |  | 9 | OTU3663(4.82%) | 1 | OTU7005(47.05%) | 1 |  |
|  |  |  |  |  |  | OTU87695(24.25%) |  |  |  |  |
| T1 | 3 | OTU182(12.71%) | 2 |  | 25 | OTU87695(30.02%) | 1 |  |  |  |
|  |  | OTU86859(47.97%) |  |  |  |  |  |  |  |  |
| T2 | 13 | OTU182(25.30%) |  |  | 21 | OTU87695(1.88%) | 1 |  | 2 |  |
|  |  | OTU86859(71.12%) |  |  |  |  |  |  |  |  |
| T3 | 16 | OTU182(83.67%) |  |  | 21 | OTU87695(1.62%) | 2 |  |  |  |
|  |  | OTU28984(2.45%) |  |  |  |  |  |  |  |  |
| T4 | 10 | OTU182(8.47%) |  |  | 21 | OTU14510(1.20%) | 2 |  |  |  |
|  |  |  |  |  |  | OTU87695(69.51%) |  |  |  |  |
| T5 | 1 |  | 1 |  | 21 | OTU87695(28.77%) | 1 | OTU7005(27.83%) | 2 |  |

Table S1 Relative abundance of dominant OTU types in each sample

* indicates spider samples used in this study, ** indicates several OTU types that exist in each kind of endosymbiont (“N” indicates the number of OTU types, and only OTU types with a relative abundance more than 1% of the whole sequence were shown in the “types of OTU”).

Table S2 The differences test of 15 kind of bacteria in different spider samples

| Taxon | *P*-Value^*^ | | | | | | | | | | | | | | | | | | | | | | | | | | | |
| --- | --- | --- | --- | --- | --- | --- | --- | --- | --- | --- | --- | --- | --- | --- | --- | --- | --- | --- | --- | --- | --- | --- | --- | --- | --- | --- | --- | --- |
|  | M-D | M-K | M-O | M-P | M-R | M-S | M-T | D-K | D-O | D-P | D-R | D-S | D-T | K-O | K-P | K-R | K-S | K-T | O-P | O-R | O-S | O-T | P-R | P-S | P-T | R-S | R-T | S-T |
| *Pseudomonas* | 0.111 | 0.016 | 0.016 | 0.343 | 0.032 | 0.343 | 0.556 | 0.548 | 0.690 | 0.730 | 0.310 | 0.556 | 0.157 | 0.841 | 0.730 | 0.690 | 0.190 | 0.056 | 0.556 | 0.690 | 0.286 | 0.056 | 0.413 | 0.886 | 0.413 | 0.190 | 0.056 | 0.905 |
| *Sphingomonas* | 0.016 | 0.016 | 0.016 | 0.029 | 0.016 | 0.029 | 0.016 | 0.841 | 0.841 | 0.556 | 0.310 | 0.905 | 1.000 | 0.690 | 0.413 | 0.548 | 1.000 | 0.841 | 0.286 | 0.421 | 0.905 | 0.690 | 0.111 | 0.486 | 0.730 | 0.413 | 0.310 | 0.905 |
| *Acinetobacter* | 1.000 | 0.190 | 0.286 | 0.686 | 0.111 | 0.343 | 0.413 | 0.151 | 0.222 | 0.730 | 0.056 | 0.063 | 0.690 | 1.000 | 0.286 | 0.310 | 0.016 | 0.690 | 0.286 | 0.690 | 0.016 | 0.548 | 0.111 | 0.057 | 0.556 | 0.016 | 0.421 | 0.063 |
| *Novosphingobium* | 0.111 | 0.016 | 0.016 | 0.686 | 0.016 | 0.114 | 0.111 | 0.548 | 0.056 | 0.190 | 0.016 | 0.556 | 0.310 | 0.310 | 0.032 | 0.095 | 0.905 | 1.000 | 0.016 | 0.841 | 0.063 | 0.310 | 0.016 | 0.200 | 0.190 | 0.111 | 0.310 | 0.905 |
| *Aquabacterium* | 0.032 | 0.016 | 0.016 | 0.486 | 0.016 | 0.114 | 0.111 | 0.421 | 0.548 | 0.413 | 0.421 | 0.190 | 0.690 | 0.690 | 0.063 | 0.690 | 0.032 | 0.421 | 0.111 | 0.690 | 0.111 | 0.548 | 0.063 | 1.00 | 0.556 | 0.016 | 0.222 | 0.730 |
| *Methylobacterium* | 0.016 | 0.016 | 0.016 | 0.029 | 0.016 | 0.029 | 0.016 | 0.548 | 1.000 | 0.556 | 0.056 | 0.730 | 0.690 | 0.548 | 0.190 | 0.421 | 0.730 | 0.841 | 0.413 | 0.095 | 0.730 | 0.841 | 0.111 | 0.343 | 0.556 | 0.413 | 0.310 | 0.905 |
| *Brevundimonas* | 0.016 | 0.016 | 0.016 | 0.029 | 0.016 | 0.029 | 0.016 | 0.151 | 0.151 | 0.111 | 1.000 | 0.413 | 0.151 | 0.841 | 0.413 | 0.143 | 0.413 | 0.310 | 0.556 | 0.206 | 0.413 | 0.310 | 0.175 | 0.886 | 0.905 | 0.175 | 0.143 | 0.905 |
| *Rhizobium* | 0.016 | 0.016 | 0.016 | 0.029 | 0.016 | 0.029 | 0.016 | 0.548 | 0.833 | 0.976 | 0.238 | 0.905 | 0.690 | 0.286 | 1.000 | 0.142 | 0.730 | 0.841 | 0.563 | 0.683 | 0.262 | 0.206 | 0.206 | 0.486 | 0.556 | 0.103 | 0.095 | 0.905 |
| *Citrobacter* | 0.698 | 0.286 | 0.286 | 0.657 | 0.167 | 1.000 | 1.000 | 0.286 | 0.286 | 1.000 | 0.167 | 0.905 | 0.651 | 1.000 | 0.405 | 1.000 | 0.246 | 0.286 | 0.405 | 1.000 | 0.127 | 0.167 | 0.167 | 0.657 | 0.492 | 1.000 | 0.167 | 0.905 |
| *Bradyrhizobium* | 0.016 | 0.063 | 0.016 | 0.114 | 0.032 | 0.057 | 0.032 | 0.151 | 0.841 | 0.190 | 1.000 | 0.190 | 0.841 | 0.151 | 0.556 | 0.310 | 0.730 | 0.421 | 0.413 | 1.000 | 0.286 | 0.841 | 0.190 | 0.686 | 0.413 | 0.413 | 1.000 | 0.556 |
| *Arthrobacter* | 0.111 | 0.016 | 0.016 | 0.343 | 0.016 | 0.029 | 0.063 | 0.056 | 0.421 | 0.730 | 0.222 | 0.902 | 1.000 | 0.222 | 0.111 | 1.000 | 0.190 | 0.151 | 0.286 | 0.421 | 0.730 | 0.690 | 0.286 | 0.486 | 0.730 | 0.413 | 0.222 | 1.000 |
| *Pseudonocardia* | 0.016 | 0.016 | 0.016 | 0.343 | 0.016 | 0.029 | 0.016 | 1.000 | 0.825 | 0.016 | 0.524 | 0.714 | 0.413 | 1.000 | 0.056 | 0.683 | 0.730 | 0.556 | 0.016 | 0.365 | 0.563 | 0.690 | 0.016 | 0.057 | 0.063 | 0.302 | 0.175 | 0.978 |
| *Microbacterium* | 0.190 | 0.016 | 0.111 | 0.343 | 0.016 | 0.200 | 0.190 | 0.016 | 0.151 | 0.730 | 0.008 | 0.413 | 0.548 | 0.310 | 0.111 | 0.151 | 0.111 | 0.421 | 0.111 | 0.690 | 0.111 | 0.222 | 0.016 | 0.200 | 0.413 | 0.016 | 0.095 | 1.000 |
| *Lactobacillus* | 0.190 | 0.167 | 0.190 | 0.314 | 0.183 | 0.314 | 0.056 | 0.690 | 0.548 | 0.278 | 0.143 | 0.262 | 0.413 | 0.841 | 0.278 | 0.214 | 0.262 | 0.690 | 0.278 | 0.214 | 0.500 | 0.413 | 1.000 | 0.714 | 1.000 | 0.921 | 1.000 | 0.921 |
| *Lactococcus* | 1.000 | 1.000 | 1.000 | 1.000 | 0.444 | 1.000 | 1.000 | 1.000 | 1.000 | 0.722 | 1.000 | 0.722 | 1.000 | 1.000 | 0.722 | 0.722 | 0.722 | 1.000 | 0.722 | 0.722 | 0.722 | 1.000 | 1.000 | 1.000 | 0.444 | 1.000 | 0.444 | 0.444 |

^*^Nonparametric Kruskal-Wallis test was used.
